# Civil society activism for Africa’s vaccine and local manufacturing agenda: Can mpox be the test case?

**DOI:** 10.1371/journal.pgph.0003738

**Published:** 2024-09-17

**Authors:** Nicaise Ndembi, George Kimathi, Achal Prabhala, Jackline Kiarie

**Affiliations:** 1 Africa Centres for Disease Control and Prevention (Africa CDC), Executive Office, Addis Ababa, Ethiopia; 2 AMREF Institute of Capacity Development, Amref International University (AmIU), Nairobi, Kenya; 3 Achal Prabhala – Accessibsa, Shuttleworth Foundation, Bangalore, India; 4 Jackline Kiarie – AMREF Health Africa, Nairobi, Kenya; PLOS: Public Library of Science, UNITED STATES OF AMERICA

In the current global health architecture, civil society organizations (CSOs) are the unsung heroes and relentless advocates of equitable access to essential medicines, medical devices and health supplies, and life-saving commodities. Civil society broadly encompasses non-governmental and not-for-profit organizations such as unions and professional organizations and networks representing their members’ interests and values [1]. As the spotlight shifts to the urgent need for local manufacturing of medical counter measures diagnostics, vaccines, medications, and other health technologies on the African continent [2], it’s high time we recognize that the table is incomplete without the invaluable presence of CSOs.

## Why is it critical to meaningfully engage CSOs?

Today, Africa battles over 100 health emergencies caused by infectious diseases annually [3]. The Africa Centres for Disease Control and Prevention, following a continental risk ranking assignment, categorized approximately 20% of these infectious diseases as high-priority vaccine-preventable diseases requiring urgent investments in, among other things, production of medical countermeasures to guarantee the health security of the African people [4] This situation represents new threats with the same old struggles—a greater need for medical diagnostics, therapeutics, and vaccines with no guarantee for increased capacity to manufacture them where they are most needed.

As HIV cases surged in the mid-1990s, particularly in Africa, the cost of antiretroviral drugs was prohibitive, making treatment inaccessible and inequitable. It is estimated that, between 1997 and 2007, 12 million HIV-positive Africans died waiting for enough life-saving drugs to reach the continent. The efforts of the US President’s Emergency Plan for AIDS Relief, the Global Fund to Fight HIV/AIDS, Tuberculosis and Malaria, CSOs, and networks of people living with HIV and other advocacy groups helped achieve price reductions, improve access and facilitated the production by multinationals of generic medicines. This set a precedent for malaria and tuberculosis health programs that incorporated free access to drugs for vulnerable populations in low-middle-income countries [5].

The COVID-19 pandemic reignited these old struggles of access to essential medicines and life-saving commodities, including vaccines, drugs, and diagnostics. Despite the optimism created by the timely development of several COVID-19 vaccine candidates globally, intellectual property barriers and nationalism led to inequities in global distribution at the height of the pandemic. Because of this, most high-income countries had achieved the World Health Organization’s target of vaccinating 70% of their populations in time, while only 32% of populations in African countries had completed their primary series by Dec 2023, nearly three years after the availability of vaccines [6].

The International Declaration of Human Rights acknowledges civil society’s role in promoting healthcare access. CSOs lead public education campaigns, build community trust, and participate in local production initiatives as was witnessed during the recent malaria vaccine introduction in Cameroon, Ghana, Kenya, and Malawi [7]. Social mobilization and community engagement by CSOs was critical for vaccine demand creation among caregivers, key opinion leaders, and local and religious leaders. Civil society also raises awareness about the importance of local production and advocates for government policies and funding to support local manufacturing. For example, the African Medicines Agency Treaty Alliance (AMATA) was set up to advocate for the ratification and implementation of the African Medical Agency (AMA). AMATA has a Steering Committee comprising member representatives, patient groups such as the International Association of Patient Organizations (IAPO), civil society organizations, industry associations, research and academic networks, and youth and advocacy groups. Amref Health Africa is a member of the AMATA steering committee and has worked very closely with the other stakeholders in advocating for the urgent ratification of the AMA treaty and its operationalization to strengthen the regulatory environment of medical products and devices on the continent. For example, AMATA advocated for enhanced market surveillance, centralised information collection and sharing of data between countries to combat falsified and substandard medicinal products on the African continent following reports by WHO that between 2013 and 2017, 42% of all fake medicines reported to them were from Africa [8]. This emphasizes the critical voice of civil society in pushing for sustainable local production and ensuring appropriate policies are implemented to protect human rights and safety.

## Great strides, clearer path ahead

That said, challenges abound. Local production is still a relatively new technical discussion at global, regional and national levels in most LMICs. This implies that CSOs may lack the skills and tools to meaningfully engage with other stakeholders on the local manufacturing landscape. Because of the complex nature of civil society interests and agendas, not to mention the complexity of this subject matter, properly coordinated engagement platforms that facilitate a collective voice in their participation in this agenda are still missing. The ability of CSOs to influence the quality of health care and its related policies depends on the availability of platforms and the willingness of partners among political or government leadership to engage [9].

Additionally, civil society encounters hurdles in maintaining long-term engagement due to their dependence on time- and budget-constrained interventions from development partners and host governments [10]. The level of civil society involvement evident today in pandemic agreement negotiation indicates that the time is ripe for the formalization of its pivotal role in the leadership and governance of local production conversations for sustainability. Governance platforms must build mutual trust between civil society and other stakeholders, creating an environment where governments provide information on manufacturing priorities and solicit public feedback through CSOs. These platforms must also allow manufacturers to engage civil society and seek support in influencing regulations and policies that support and incentivize local production of medical countermeasures. These include favourable tax incentives, supportive intellectual property policies, and regulatory reforms to promote local production. Many CSOs have also called for the right to access essential medicines to be embedded in the pandemic treaty which is an opportunity to promote effective and equitable access to medical countermeasures [11].

## Looking forward—Strengthening civil society’s capacity to engage

It is time to recognize the indispensable role of civil society and usher in an era of collaborative and inclusive strategies that will secure a healthier, more resilient future for us all. To enhance the voices of CSOs, governance platforms should facilitate the building of mutual trust between CSOs and stakeholders, enabling governments to share manufacturing priorities and gather public input; and support local manufacturers in engaging with CSOs to influence policies and regulations that promote local production, such as favorable tax incentives and regulatory reforms. These platforms should also facilitate continuous capacity-building for CSOs to effectively advocate for sustainable local production, contributing to a more resilient and self-reliant healthcare infrastructure in Africa.
